# Prohormone convertase 2 activity is increased in the hippocampus of Wfs1 knockout mice

**DOI:** 10.3389/fnmol.2015.00045

**Published:** 2015-08-25

**Authors:** Karin Tein, Sergo Kasvandik, Sulev Kõks, Eero Vasar, Anton Terasmaa

**Affiliations:** ^1^Department of Physiology, Institute of Biomedicine and Translational Medicine, University of TartuTartu, Estonia; ^2^Proteomics core facility, Institute of Technology, University of TartuTartu, Estonia; ^3^Department of Pathophysiology, Institute of Biomedicine and Translational Medicine, University of TartuTartu, Estonia; ^4^Department of Reproductive Biology, Estonian University of Life SciencesTartu, Estonia

**Keywords:** Wfs1, prohormone convertase 2, peptidomics, mass-spectrometry, proSAAS, 7B2, peptide processing

## Abstract

**Background:** Mutations in WFS1 gene cause Wolfram syndrome, which is a rare autosomal recessive disorder, characterized by diabetes insipidus, diabetes mellitus, optic nerve atrophy, and deafness. The WFS1 gene product wolframin is located in the endoplasmic reticulum. Mice lacking this gene exhibit disturbances in the processing and secretion of peptides, such as vasopressin and insulin. In the brain, high levels of the wolframin protein have been observed in the hippocampus, amygdala, and limbic structures. The aim of this study was to investigate the effect of Wfs1 knockout (KO) on peptide processing in mouse hippocampus. A peptidomic approach was used to characterize individual peptides in the hippocampus of wild-type and Wfs1 KO mice.

**Results:** We identified 126 peptides in hippocampal extracts and the levels of 10 peptides differed between Wfs1 KO and wild-type mice at P < 0.05. The peptide with the largest alteration was little-LEN, which level was 25 times higher in the hippocampus of Wfs1 KO mice compared to wild-type mice. Processing (cleavage) of little-LEN from the Pcsk1n gene product proSAAS involves prohormone convertase 2 (PC2). Thus, PC2 activity was measured in extracts prepared from the hippocampus of Wfs1 KO mice. The activity of PC2 in Wfs1 mutant mice was significantly higher (149.9 ± 2.3%, p < 0.0001, n = 8) than in wild-type mice (100.0 ± 7.0%, n = 8). However, Western blot analysis showed that protein levels of 7B2, proPC2 and PC2 were same in both groups, and so were gene expression levels.

**Conclusion:** Processing of proSAAS is altered in the hippocampus of Wfs1-KO mice, which is caused by increased activity of PC2. Increased activity of PC2 in Wfs1 KO mice is not caused by alteration in the levels of PC2 protein. Our results suggest a functional link between Wfs1 and PC2. Thus, the detailed molecular mechanism of the role of Wfs1 in the regulation of PC2 activity needs further investigation.

## Introduction

Wolfram syndrome (WS) was first described by Wolfram and Wagener (1938) and is a rare autosomal recessive disorder characterized by diabetes insipidus (DI), diabetes mellitus (DM), optic nerve atrophy (OA), and deafness (D) – DIDMOAD (Strom et al., 1998). WS is caused by mutations in WFS1 gene, which is located in the fourth chromosome 4p16.1 region. Approximately 1% of the general population are heterozygous carriers of WFS1 gene mutation and it has been found that these carriers have increased risk for developing a psychiatric disease (Swift et al., 1991). Though not all mutations in WFS1 gene cause WS, carriers of WFS1 gene mutations have a 26 times higher risk for psychiatric diseases such as depression and bipolar disorder (Swift and Swift, 2000). Patients without WS, but exhibiting bipolar disease, major depression, and schizophrenia have been found to carry mutations in WFS1 gene (Strom et al., 1998; Evans et al., 2000; Torres et al., 2001; Crawford et al., 2002).

WFS1 gene product is wolframin, a glycoprotein of 890 amino acid residues and molecular weight of about 100 kDa (Strom et al., 1998). Wolframin is mostly found in the brain, heart and pancreatic insulin-producing β-cells. The highest levels of expression in the brain are found in the hippocampus, amygdala, allocortex and olfactory bulb (Takeda et al., 2001). On an intracellular level, Wfs1 protein is located in the membrane of the endoplasmic reticulum (ER), where it forms ∼400 kDa protein complexes (Hofmann et al., 2003). Wolframin influences cellular Ca^2+^ homeostasis and β-cell survival (Hofmann et al., 2003). In addition, Wfs1 protein takes part in the regulation of the ER-stress response (Rigoli et al., 2011).

Human studies have shown that peptide processing is altered in patients with WS. Immunohistochemistry of the autopsy samples reveal that processed vasopressin is absent in the supraoptic and paraventricular nuclei of hypothalamus in patients with WS (Gabreels et al., 1998). This was accompanied by a lack of expression of prohormone convertase 2 (PC2) and the chaperone secretogranin V (7B2) in this brain region of WS patients (Gabreels et al., 1998). In addition to its localization in the ER membrane, Wfs1 is also localized in the secretory granules of beta-cells and participates in acidification of these granules (Hatanaka et al., 2011). Conversion of proinsulin to insulin takes place in the secretory granules and is pH-dependent (Hatanaka et al., 2011), thus, lack of Wfs1 results in impaired insulin processing, which is manifested by an increased proinsulin/insulin ratio (Hatanaka et al., 2011; Noormets et al., 2011).

The role of Wfs1 in the regulation of insulin secretion and survival of pancreatic beta-cells is a topic of ongoing research and has attracted wide attention. On the other hand, there is only one report on the role of WFS1 on the processing of brain peptides (Gabreels et al., 1998). One of the regions in the brain with the highest expression of Wfs1 is hippocampus (Luuk et al., 2008). Thus, the aim of the present study was to evaluate the processing of neuropeptides in this brain region using a peptidomic approach. We have identified peptides in which processing is altered in the hippocampus of Wfs1 KO mice, and evaluated the activity of peptide processing enzymes that take part in the processing of these peptides.

## Materials and Methods

### Wfs1 Knockout Mice

Test animals were male 129Wfs1 gene mutation mice (129Wfs1KO) and control group animals were male wild-type littermate (129WT) mice. Animals were kept in polypropylene cages. The air temperature in the room was 21 ± 2°C and light cycle was 12 h light and 12 h dark. The light was switched on at 7.00 am. Chow and drinking water was available *ad libitum*. All of the procedures performed with animals were approved by the Estonian National Board of Animal Experiments (No. 86, 28.08.2007) and were in accordance with the European Union directive 86/609/EEC.

Generation of whole body Wfs1 knock out (KO) mouse was described previously (Noormets et al., 2009). Briefly, Wfs1 deficient mice were generated by replacing most of the coding region of the Wfs1 gene (exon8) with LacZ sequence. This results in the deletion of the amino acids 360–890 of the Wfs1 protein and a fusion between remaining Wfs1 fragment and LacZ. The official designation of this strain is Wfs1tm1Koks according to the Mouse Genome Database^1^. Mice with the isogenic 129S6 background were used in this study. Genotyping of the mice was performed by multiplex PCR for both alleles using specific primers as described previously (Noormets et al., 2009).

In general, eight Wfs1 KO and eight wild-type animals were used for each kind of analysis, as different tissue preparation was needed for different experiments. Altogether 96 mice were used in this study.

### Tissue Preparation

At the age of 3 months mice were sacrificed by cervical dislocation. The brain was removed and the hippocampus dissected out on a cold plate. Immediately thereafter tissue samples were frozen in liquid nitrogen and kept at -80° C until the analyses. Body weight of Wfs1 KO mice was smaller compared to wild-type littermates (22.0 ± 0.7 g and 28.3 ± 0.5 g, respectively; *p* < 0.0001; *n* = 30).

### Peptide Extraction

Peptides were extracted from hippocampal tissue by a double extraction protocol based on Frese et al. (2013). One hundred fifty microliter of acidified acetone (acetone/water/concentrated HCl 40:6:1) was added to the tissue sample. After microtip sonication, samples were incubated for 1 h on ice. The samples were then centrifuged for 25 min at 14,000 *g* (4°C), the supernatant was transferred to a new tube and neutralized by adding 1 M NaOH (1:1 ratio to HCl). Acetone was evaporated using a vacuum centrifuge. The pellet was washed with 0.25% acetic acid, incubated on ice for an hour and collected by centrifugation for 25 min at 14,000 *g* (4°C). The pellet and the supernatant were combined and filtered through a Millipore 10 kDA Amicon Ultra YM-10 membrane for 30 min at 7,000 g. The filtered peptide extract was desalted using C18 StageTips (Rappsilber et al., 2007) and reconstituted in 0.5% TFA.

### Nano-LC/MS/MS Analysis

**FIGURE 1:**
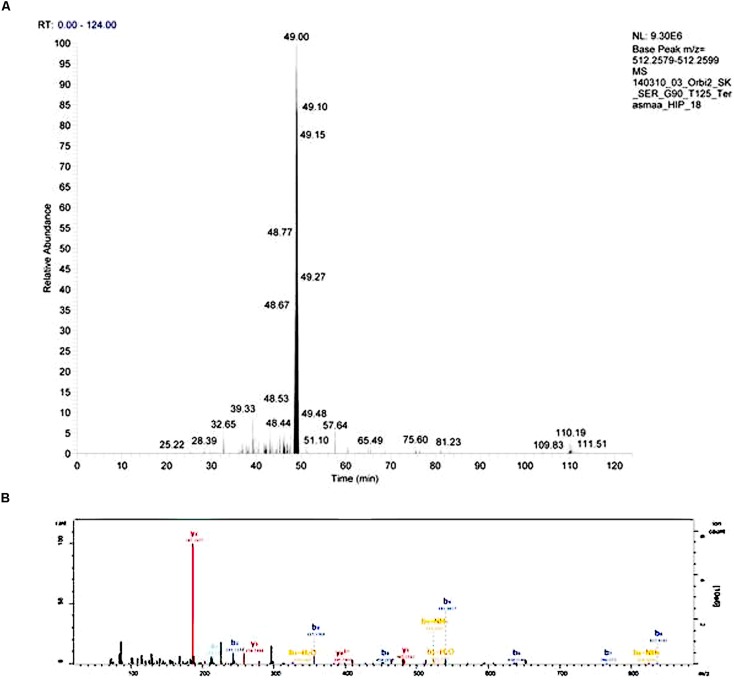
**An example of an extracted chromatogram **(A)** and MS/MS spectrum **(B)** of peptide LENPSPQAPA (little-LEN).** The b- and y-series ions are annotated on the MS/MS spectrum.

Injected peptides were separated on Ultimate 3000 RSLCyano system (Dionex) using a C18 cartridge trap-column in backflush configuration and an in-house packed (3 μm C18 material, Dr MaischGmbh) analytical 50 cm × 75 μm emitter-column (New Objective). Peptides were eluted at 200 nl/min with a 8–40% B 90 min gradient (buffer B: 80% ACN + 0.1% FA, buffer A: 0.1% FA) to a Q Exactive (Thermo Fisher Scientific) tandem mass spectrometer operating with a top-10 strategy and a cycle time of 0.9 s. Briefly, one 350–1400 m/z MS scan at a resolution setting of *R* = 70,000 was followed by higher-energy collisional dissociation fragmentation (normalized collision energy of 25) of 10 most intense ions (>+1 charge state) at *R* = 17,500. Dynamic exclusion was limited to 40 s. An example of chromatogram and mass spectrum are presented on **Figure 1**.

### Raw Data Analysis

Raw data was processed with MaxQuant 1.4.0.8 software package (Cox and Mann, 2008). Methionine oxidation and protein N-terminal acetylation were set as variable modifications. Search was performed against UniProt^2^ mouse neuropeptides database (March 2014) using unspecific digestion rule. MS and MS/MS mass errors were 4.5 and 20 ppm, respectively. Only peptides minimally six amino acids long were accepted and transfer of identifications between runs was allowed. Peptide-spectrum match and protein FDR was kept below 1% using a target-decoy approach. All other parameters were default.

Raw intensity values for each sample were summed up and intensity value for each peptide in this sample was normalized for the sum. Normalized values were analyzed using Student’s *t*-test. *P*-value of less than 0.05 was considered as statistically significant.

### Enzymatic Activity of PC2

The method is based on measuring the liberation of aminocoumarin using a fluorescence spectrophotometer developed by Li et al. (2003). Aminocoumarin is cleaved from the fluorogenic substrate pGlu-Arg-Thr-Lys-Arg-AMC via PC2-mediated cleavage. Hippocampus was weighed and sonicated in 1 mL lysis buffer (0.1 M sodium acetate pH 5.5; 1% TritonX-100, 1 μM E64 (Sigma–Aldrich), 1 μg/ml pepstatin (Sigma–Aldrich), and 1 mM phenylmethylsulfonyl fluoride (PMSF). The volume of buffer was adjusted to give tissue concentration 10 mg/ml. After centrifugation for 10 min at 10,000 *g*, supernatant was used for analysis of enzyme activity. Samples and reagents were kept on ice at all times. Test tubes were prepared for the measurement of enzymatic activity with final assay volume 200 μL, containing 50 μL supernatant, 0,1 M sodium acetate (pH 5.5), and 2.5 mM calcium chloride and 0.25% TritonX-100. In order to test specificity, the synthetic peptide (7B2-CT), which corresponds to the C-terminal portion of human 7B2 (residues 155–185) was added to half of the tubes at 30 μM final concentration and tubes were incubated for 15 min at 37°C. Then the substrate p-Glu-Arg-Thr-Lys-Arg-AMC (Sigma–Aldrich, final concentration 0.2 mM) was added to all tubes. Substrate was first dissolved in DMSO; the final concentration of DMSO in the reaction mix was 2%. The reaction was 17 h long at 37°C. The fluorescence of disengaged aminomethylcoumarin was measured using fluorescence – luminescence spectrophotometer (Luminescence Spectophometer LS50B, Perkin Elmer) with excitation at 380 nm and emission at 460 nm. Control samples without enzyme and substrate were used as blanks. Specific PC2 activity was calculated as difference of total activity of the reaction and the activity in the presence of 7B2-CT peptide. Activity in the presence of 7B2-CT was approximately 25–30% of total activity.

### Enzymatic Activity of Carboxypeptidase E (CPE)

The method is based on the cleavage of the terminal arginine from the substrate using a fluorescence spectrophotometer developed by Fricker et al. (1996). Hippocampus was dissected, weighed, and homogenized in 2 ml lysis buffer (0.1 M NaAc, pH 5.5, 1 mM PMSF). For measuring carboxypeptidase E (CPE) activity, 25 μL of the homogenate was added to 50 mM NaAc buffer (pH 5.5), containing 0.01% Triton X100 and 200 μM dansyl – Phe – Ala – Arg substrate in final volume of 250 μL. In addition, reaction mix contained either 1 mM CoCl_2_ or 1 μM guanidinoethylmercaptosuccinic acid (GEMSA). After incubation at 37°C for 30 – 60 min, 100 μL of 0.5 M HCl was added to stop the reaction and 2 mL of chloroform was added to extract dansyl – Phe – Ala. Tubes were mixed and centrifuged at 500 × *g* for 2 min. The amount of product was determined by measuring the fluorescence (excitation 350 nm, emission 500 nm) in the chloroform layer. CPE activity is defined as the difference between activity measured in the presence of Co^2+^ (activates CPE) and in the presence of GEMSA (inhibits CPE). Non- specific fluorescence was measured in the absence of the tissue extract. All measurements were done in duplicates.

### Western Blot Analysis

Western Blot analysis for PC2 was done using NuPAGE system (Life Technologies) according to manufacturer’s Membranes with transferred proteins were blocked for 1 h in 3% BSA in PBS. Thereafter, the membranes were incubated with primary antibody overnight at 4°C and were rinsed six times in Milli-Q water. Membranes were incubated with secondary antibody for 1 h at room temperature and washed six times in Milli-Q water. Membranes were washed once for 20 min in PBS containing 0.1% Tween-20. Antibody detection was performed using the LI-COR Odyssey CLx system (LI-COR Biotechnologies). Images were converted to grayscale and quantification was performed using Image Studio Lite v 3.1.4 (LI-COR Biotechnologies). A commercial anti-PC2 antibody (Thermo-Scientific, dilution 1:200) was used to detect active isoform of PC2 (mass 69 kDa) and proPC2 (mass 72 kDa). The secondary antibody was Alexa-790 conjugated donkey anti rabbit (Jackson ImmunoResearch Laboratories, Inc., dilution 1:40,000).

### RNA Isolation, cDNA Synthesis and Quantitative Real-Time PCR

Samples were put on ice and homogenized with 200 μL Trizol (Trizol^®^ Reagent, Invitrogen, USA) when the tissue was slightly thawed. Samples were incubated at room temperature for 5 min, chloroform was added and tubes were shaken for 15 sec. Samples were again incubated for 2 min at room temperature. Samples were centrifuged for 15 min, 12,000 *g* at 4°C. The upper water phase was removed to a new 1.5 mL tube and isopropanol in half of the Trizol volume was added. Tubes were turned to mix carefully and incubated for 10 min at room temperature. Samples were centrifuged for 10 min, 12,000 *g* at 4°C. Supernatant was taken away carefully and 1 mL of ice-cold 75% EtOH was added to RNA. Tubes were centrifuged for 10 min, 10,000 *g*, at 4°C. Supernatant was removed carefully and tubes were left to dry until all the EtOH was evaporated. 250 μL MQ was added and RNA was frozen at -80°C. Before freezing the samples, RNA quality control was performed using Nanodrop. The 260/230 and 260/280 ratios were around 2.00.

One microgram of total RNA of each sample with random hexamers (Applied Biosystems) and SuperScript^TM^ III Reverse Transcriptase (Invitrogen, USA) were used for the first strand cDNA synthesis. Thermal cycling was performed using 7900HT Fast Real- time PCR system (Applied Biosystems) at 95°C for 10 min, then 40 cycles at 95°C for 15 sec and 60°C for 1 min. Probes were designed from exon–exon junctions eliminating the possibility of genomic DNA contamination. Hprt1 was used as housekeeper gene as it is a reliable reference gene.

For RT-PCR, each sample was performed in four parallel reactions. The experiment was repeated two times and a negative control was used. The amplification curves of the housekeeping gene, PC2 and 7B2 were similar and the amount for each transcript was calculated using standard curve of cycle threshold for serial dilutions of the sample and normalized to Hprt1 expression.

### Radioimmunoassay (RIA)

Acid extracts (0.1 M HCl) were prepared from tissues by homogenizing in 200 (pituitary) or 1 ml (hippocampus) of ice-cold 0.1 M HCl. The samples were centrifuged at 15,000 *g* for 15 min and the clear supernatant was lyophilized. Samples were reconstituted in 0.5 ml of RIA buffer (-0.1 M sodium phosphate, pH 7.4, with 50 mM sodium chloride, and 0.1% BSA and azide), shaken for 30 min at room temperature, centrifuged, and 100 μL duplicates measured for 7B2 using a sensitive RIA employing an antiserum to residues 23–39 (Zhu and Lindberg, 1995).

### Protein Measurement

Micro BCA^TM^ Protein Assay Kit (Thermo Scientific) was used for the protein measurement and the analysis was done according to the manufacturer’s protocol.

### Statistical Analysis

Unpaired two-tailed Student’s *t*-test was used to compare experimental groups. A *P*-value of less than 0.05 was considered as statistically significant. Data are presented as mean ± SEM.

## Results

### Peptidomic Study

One hundred and twenty-six individual peptides with sequence length of 8–24 amino acids were identified (Supplementary Table S1). The molecular weight of identified peptides was in the range from 757 to 2817 Da. Most of the identified peptides were derived from 11 precursor sequences: galanin; orexin/hypocretin; neuropeptide Y; proprotein convertase subtilisin/kexin type 1 inhibitor; preprodynorphin; proenkephalin-A; melanin-concentrating hormone; prepronociceptin; neuroendocrine protein 7B2; substance P and teneurin-1.

**FIGURE 2:**
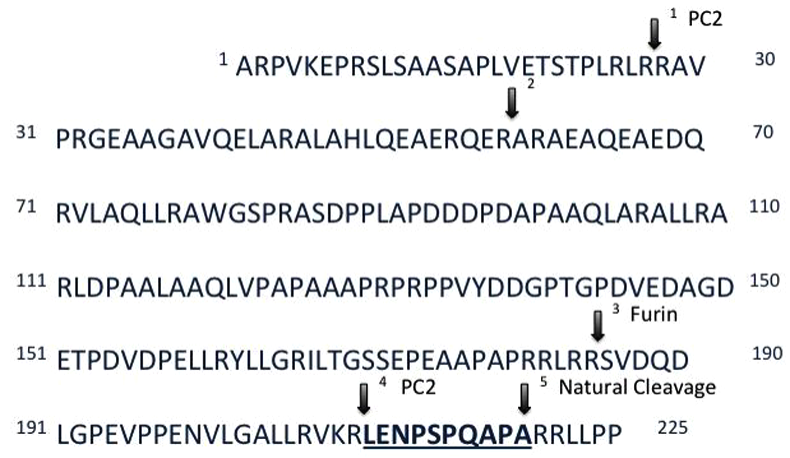
**Primary sequence and processing sites of proSAAS**. Prohormone convertase 2 (PC2) participates in the cleavage of proSAAS at residue 209 (arrow 4). The amount of the underlined peptide little-LEN is 25 times higher in the hippocampus of the Wfs1 KO mice as compared to their wild-type littermates, which prompted the measurement of PC2 activity in Wfs1 KO mice.

The levels of 10 peptides were found to be different in Wfs1 KO versus wild-type mice (**Table 1**). The peptide with the biggest fold change was little-LEN, a peptide derived from proSAAS. Little-LEN is cleaved from proSAAS in a PC2 dependent manner (**Figure 2**). As the level of little-LEN was 25 times higher in Wfs1 KO mice, we decided to measure the activity of PC2 in the hippocampus of these mice.

**Table 1:** The list of identified peptides from mice hippocampus whose level was dependent on the Wfs1 genotype (*n* = 6 per genotype).

Precursor gene name	Peptide sequence	Mass, Da	Measurement error, ppm	Peptide name	Fold change Wfs1 KO/wild-type	*p*-value
Pcsk1n	LENPSPQAPA	1022.503	-0.42	Little-LEN	25.19	0.0016
Penk	YGGFMRSL	929.4429	-1.08	Met-enkephalin-Arg-Gly-Leu	4.98	0.0065
	LRGNKSIS	873.5032	-0.82		3.05	0.0069
Pcsk1n	SLSAASAPLVETSTPL	1542.814	-0.15	Little-SAAS 1–16	0.69	0.011
	DSDSEAYPEDSERR	1654.671	0.38		1.47	0.018
Pcsk1n	SVDQDLGPEVPPENVLGAL	1947.979	-0.94	PEN-19	0.32	0.027
Pcsk1n	SVDQDLGPEVPPENVLGALLRV	2316.233	-4.33	PEN	0.22	0.036
Pcsk1n	SVDQDLGPEVPPENVLGA	1834.895	-0.84	PEN-18	0.56	0.040
Scg5	VEYPAHQAMNLVGPQSIEG	2038.978	-1.46		0.52	0.041
Penk	PEWWMDYQ	1153.454	-0.62	Peptide-E	0.40	0.05

### Prohormone Convertase 2 Activity Measurement

**FIGURE 3:**
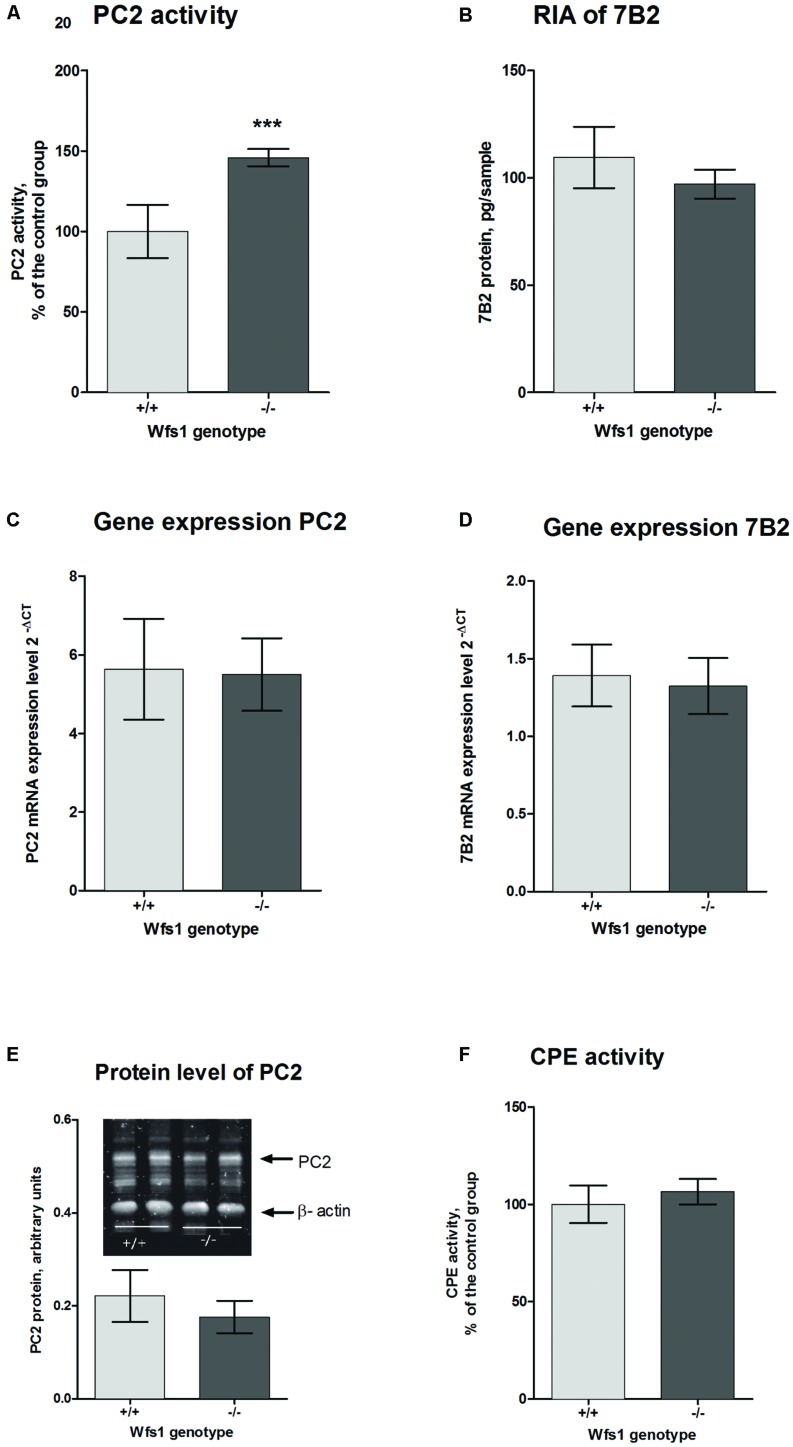
**Prohormone convertase 2 in the hippocampus of Wfs1 mice. (A)** PC2 activity is higher in Wfs1 KO mice (149.9 ± 2.3%, *n* = 8, ^∗∗∗^*p* < 0.0001) than wild-type mice (100.0 ± 7.0%, *n* = 8). The activity of PC2 is shown as percentage of the wild-type group. PC2 activity in the hippocampus of wild-type mice was 12.1 pmol/mg protein/min. Activity in the presence of 7B2-CT was approximately 25–30% of total activity. **(B)** Radioimmunoassay (RIA) for measuring 7B2 protein level. Immunoreactivity was slightly higher in wild-type mice (109.5 ± 6.0, *n* = 8) than in Wfs1 KO mice (97,1 ± 2,9, *n* = 8), though these results are not significantly different. **(C,D)** mRNA levels of PC2 and 7B2, respectively, were measured with qPCR. PC2 mRNA expression levels were similar in both groups (5.63 ± 0.53, *n* = 7 in wild-type mice and 5.50 ± 0.39, *n* = 8 in Wfs1 KO mice). 7B2 mRNA expression levels were similar in both groups (1.39 ± 0.08, *n* = 8 in wild- type mice and 1.32 ± 0.08, *n* = 8 in Wfs1 KO mice). **(E)** Protein levels of PC2 were similar in Wfs1 KO mice (0.77 ± 0.14, *n* = 8, *p* = 0.27) and in wild-type mice (1.00 ± 0.10, *n* = 8). **(F)** Carboxypeptidase E (CPE) activity was similar in Wfs1 KO mice (106.5 ± 2.7%, *N* = 8) and wild-type mice (100.0 ± 4.01%, *n* = 8). Student’s *t*-test was used for statistical analysis.

The activity of PC2 in the hippocampus of Wfs1 KO mice was measured using a fluorogenic substrate coupled with a specific PC2 inhibitor. PC2 activity was significantly higher in Wfs1 KO mice compared to wild-type mice (**Figure 3A**), which is in accordance with the elevated little-LEN peptide levels in Wfs1 KO mice. PC2 participates in the cleavage of proSAAS at residue 209 (Sayah et al., 2001), thus producing little-LEN (**Figure 2**, arrow 4).

As the activity of PC2 was significantly higher in Wfs1 KO animals, we measured the protein and mRNA levels of PC2 as well to see whether the increased activity was due to elevated mRNA or protein levels. 7B2 performs a dual function for PC2: while the C-terminal part of 7B2 inhibits active PC2, the remainder of the protein is needed to obtain active PC2 from proPC2. Therefore, we also measured mRNA and protein levels of 7B2.

### PC2 and 7B2 Protein and mRNA Levels

Prohormone convertase 2 and 7B2 protein levels were measured with Western blot analysis. There was no difference in PC2 protein levels between the groups (**Figure 3E**). As Western blotting is not sensitive enough to measure 7B2 protein levels, RIA was used for that purpose. There was no difference in 7B2 protein levels in Wfs1 KO and wild-type mice (**Figure 3B**). There was also no difference in mRNA levels of PC2 and 7B2 (**Figures 3C,D**).

In sum, we found no significant difference in the protein and mRNA levels of both PC2 and 7B2, yet the activity of PC2 was significantly higher and so were the levels of peptide little-LEN, a product thought to be generated by PC2.

### CPE Activity

To obtain active neuropeptides and peptide hormones, two sets of enzymes are needed. First, PCs, like PC2, cleave the precursor at specific sites to generate peptides with C-terminal basic residues; these intermediates are then cut by CPE to remove basic residues (Fricker et al., 1996). Therefore we measured the activity levels of CPE with fluorescence spectrophotometry. We found no significant difference of CPE activity levels in the hippocampus of Wfs1 KO (106.5 ± 2.7%, *N* = 8) and wild-type mice (100.0 ± 4.01%, *N* = 8, **Figure 3F**).

## Discussion

The Wfs1 protein is highly expressed in pancreatic beta-cells, and the role of Wfs1 in the processing and secretion of insulin is relatively well studied (Hatanaka et al., 2011). Wfs1 is also expressed in the brain, and the highest levels of this protein are found in the limbic structures, amygdala, second layer of the cortex and hippocampus (Luuk et al., 2008). Heterozygous mutations in *WFS1* increase the risk of psychiatric disorders without causing WS (Swift et al., 1998; Swift and Swift, 2000, 2005), although this view has been challenged (Kato et al., 2003; Martorell et al., 2003). Nevertheless, a recent meta-analysis of genome-wide expression studies revealed *WFS1* mRNA to be significantly elevated in the prefrontal cortex of patients with bipolar disorder (Seifuddin et al., 2013). Therefore, Wfs1 seems to be important in the regulation of brain function in addition to its well-known role in neurodegeneration. To the best of our knowledge, there is only one study connecting WFS1 with peptide processing in the brain, in which lack of vasopressin processing was revealed in the hypothalamus of WS patients (Gabreels et al., 1998). Therefore, the aim of this study was to evaluate the peptide profile in the hippocampus of Wfs1 KO mice using a peptidomic approach. The hippocampus was chosen as this region shows high expression levels of Wfs1 (Luuk et al., 2008).

In this study we used mice of the age of 3 months. At this age Wfs1 KO animals already display characteristic phenotype. The growth of Wfs1 KO mice stops at age of 2 months while wild-type mice continue to grow, thus, body weight of Wfs1 KO animals is smaller than body weight of wild-type animals at the age of 3 months (Luuk et al., 2009; Punapart et al., 2014). Wfs1 KO mice used in this study are also smaller than their wild-type littermates, indicating functional consequence of Wfs1 invalidation. The basal blood glucose levels are similar for both genotypes even at the age of 6 months (Terasmaa et al., 2011), while Wfs1 KO animals display glucose intolerance already at the age of 2–3 months (Luuk et al., 2009). Although glucose tolerance in Wfs1 KO mice was not measured in this study, we can presume that 3 months old Wfs1 KO mice used in this study display glucose intolerance that reflect a lack of Wfs1 function. WS is a severe disorder that is characterized by optic atrophy, deafness, and diabetes (Rigoli et al., 2011). Glucose intolerance of Wfs1 KO mice seem to correlate well with the human condition. However, other characteristics of WS may take longer time to manifest in mice and therefore, direct comparison of mice with patients suffering from WS is not straightforward.

A mass-spectrometric approach to identify individual peptides was used in this study. 126 individual peptides were identified and levels of 10 peptides were statistically significantly dependent on the Wfs1 genotype.

The peptide with the largest increase was little-LEN, a product of cleavage of proSAAS (the *Pcsk1n* gene product). The closely related peptide big-LEN was recently found to be an agonist for hypothalamic G protein-coupled receptor GPR171, which plays a role in central regulation of feeding and metabolism (Gomes et al., 2013). However, little-LEN has no activity at GPR171 (Gomes et al., 2013). Little-LEN is cleaved from pro-SAAS in a PC2- and furin-dependent manner (Sayah et al., 2001) (**Figure 2**), we therefore evaluated the activity of PC2 in Wfs1 KO mice. Our results indicate that PC2 activity is upregulated in the hippocampus of Wfs1 KO mice. This is in contrast with a previous report, where lack of vasopressin processing was associated with the absence of PC2- and 7B2-immunoreactivity in the paraventricular nucleus of WS patients (Gabreels et al., 1998). Also, the protein level of PC2 was not changed in the pancreatic beta-cells of Wfs1-null mice (Hatanaka et al., 2011). Thus, PC2 might be differentially regulated by Wfs1 in different cell types. In this study we found no alterations in protein or mRNA levels of PC2 and its chaperone 7B2 in the hippocampus of Wfs1 KO mice. As the activity of PC2 is increased, there is a possibility that Wfs1 regulates enzymatic activity of PC2 via a completely different mechanism rather than simply regulating its expression level.

**FIGURE 4:**
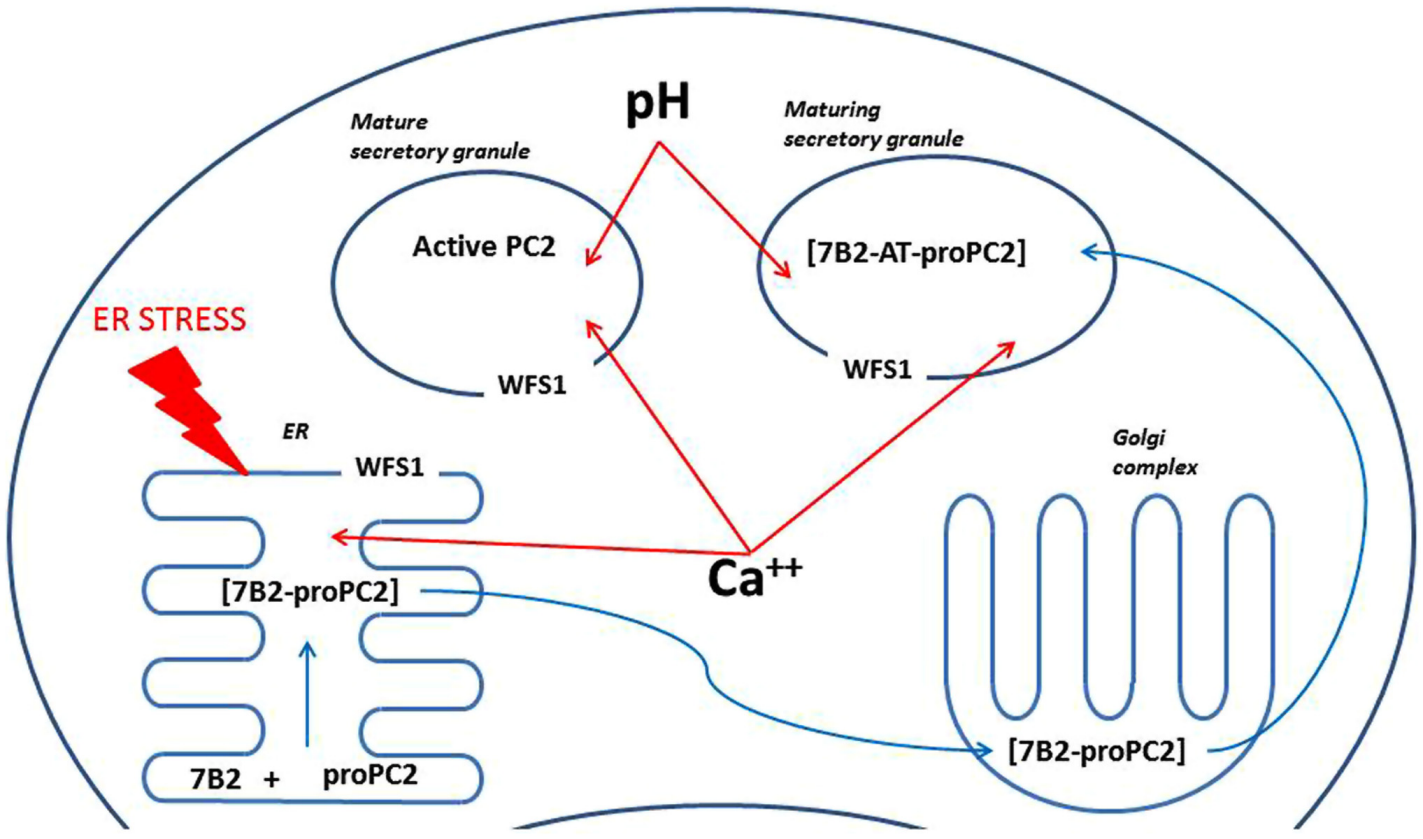
**A scheme illustrating possible mechanisms of regulation of PC2 by Wfs1**. ProPC2 with its chaperon 7B2 are transported into endoplasmic reticulum (ER), where they form a complex, which exits the ER without PC2 propeptide cleavage. Thereafter 7B2- proPC2 complex enters Golgi complex, where 7B2 is internally cleaved into 21 kDa amino terminal part (7B2-AT) and 31 amino acids long carboxy-terminal part. Then 7B2-AT-proPC2 complex is transported into maturing secretory granules. In the maturing secretory granules PC2 propeptide is cleaved to an active PC2. Wfs1 protein is localized in the membrane of ER and also secretory granules, where it regulates Ca^++^ levels and pH. In turn, pH and Ca^++^ are known to regulate activity of PC2. In addition, lack of Wfs1 function leads to increased ER stress, which can alter proper folding of proPC2 and 7B2 in the ER.

Prohormone convertase 2 is found in neuroendocrine cells, which contain a regulatory secretory pathway. These cells are capable of processing different prohormones like provasopressin, proinsulin, proenkephalin, prosomatostatin, pro-TRH, proopiomelanocortin (POMC) and many others (Benjannet et al., 1991; Breslin et al., 1993; Brakch et al., 1995; Friedman et al., 1995). The main task of PC2 is proteolytic processing of neuropeptide and peptide hormone precursors. The PCs are widely expressed in the brain cortex, hippocampus, and hypothalamus (Schafer et al., 1993). PC2 itself is activated via proteolytic cleavage; immature 75-kDa proPC2 is cut to yield active 68-kDa PC2. Such cleavage is not necessary for PC2 secretion, but is needed for its activation (Taylor et al., 1997). PC2 differs from all other PCs in its requirement for interaction with the neuroendocrine-specific protein 7B2 to generate active PC2 (Zhu and Lindberg, 1995). Interaction between proPC2 and 7B2 is complex and occurs in different cellular compartments (**Figure 4**; Muller and Lindberg, 1999). The 7B2 protein was first isolated from the pituitary gland in the 1980s (Martens, 1988). It is found in the central nervous system and endocrine tissues (Seidah et al., 1983). 7B2 is synthesized as 185 amino acid precursor protein and during passage through the secretory pathway it is cut into two fragments – amino-terminal (21-kDa) and carboxyl-terminal part (7B2-CT; Laurent et al., 2002). The amino-terminal part is necessary to activate proPC2 (Zhu and Lindberg, 1995); and at the same time, the 7B2-CT peptide is a highly selective PC2 activity inhibitor (van Horssen et al., 1995). However, we found no alterations in mRNA and protein levels of 7B2 in the hippocampus of Wfs1 KO mice (**Figure 3D**).

The activity of PC2 is further regulated in several different ways. Acidification of secretory granules is required for proper functioning of PC2 (Hatanaka et al., 2011) and acidification of secretory granules in pancreatic beta-cells is Wfs1 dependent (Hatanaka et al., 2011). However, the effect of Wfs1 invalidation on acidification of secretory granules in the brain is unknown. In addition to the pH, maturation of PC2 is also regulated by calcium levels (Shennan et al., 1995) and Wfs1 is well known to be involved in regulation of intracellular calcium (Osman et al., 2003). Also, the activity of 7B2 is regulated via its phosphorylation (Lee et al., 2006). We suggest that Wfs1 can regulate activity of PC2 via any of these mechanisms in the brain. However, the precise mechanism of involvement of Wfs1 in the regulation of PC2 activity in the brain is unknown and needs further attention. As all of the proposed mechanisms of regulation of PC2 are on the intracellular level, it is also necessary to investigate whether PC2 and Wfs1 are co-expressed in the same cells in the brain.

The peptide whose level showed the next largest Wfs1-dependent change was Met-enkephalin-Arg-Gly-Leu, a product of proenkephalin cleavage (**Table 1**). This peptide showed strongly reduced levels in the hypothalamus of PC2-KO mice (Pan et al., 2006). In this study, we found that the level of Met-enkephalin-Arg-Gly-Leu was almost five times higher in the hippocampus of Wfs1 KO mice, thus, pointing to a higher activity of PC2. A higher level of this peptide further supports the increased activity of PC2 in the hippocampus of Wfs1 KO mice.

## Conclusion

We identified 126 individual peptide fragments in mouse hippocampal extracts. Analysis of the relative abundance of these peptides revealed alterations in the processing of proSAAS and proenkephalin in the hippocampus of Wfs1 KO mice, which is most likely caused by an increase in the activity of PC2. The increased activity of PC2 in Wfs1 KO mice is not a result of increased levels of the PC2 protein or its chaperone 7B2. Thus, the role of Wfs1 in the regulation of PC2 activity requires further investigation.

## Author Contributions

KT, AT, SK, and EV conceived the study, SergoK performed mass-spectrometry analysis, KT and AT extracted peptides, measured enzymatic activity and performed Western blot analysis. SergoK, AT, and KT analyzed the data, KT and AT wrote the manuscript. All the authors have read and approved the final version of the manuscript.

## Conflict of Interest Statement

The authors declare that the research was conducted in the absence of any commercial or financial relationships that could be construed as a potential conflict of interest.

## Footnotes

1 http://www.informatics.jax.org

2 www.uniprot.org
